# Combined Therapy with Cytokine-Induced Killer Cells and Oncolytic Adenovirus Expressing IL-12 Induce Enhanced Antitumor Activity in Liver Tumor Model

**DOI:** 10.1371/journal.pone.0044802

**Published:** 2012-09-18

**Authors:** Zhi Yang, Qianzhen Zhang, Ke Xu, Juanjuan Shan, Junjie Shen, Limei Liu, Yanmin Xu, Feng Xia, Ping Bie, Xia Zhang, Youhong Cui, Xiu-wu Bian, Cheng Qian

**Affiliations:** 1 Institute of Pathology and Southwest Cancer Center, Southwest Hospital, Third Military Medical University, Chongqing, China; 2 Institute of Hepatobiliary Surgery, Southwest Hospital, Third Military Medical University, Chongqing, China; The University of Chicago, United States of America

## Abstract

Both adoptive immunotherapy and gene therapy hold a great promise for treatment of malignancies. However, these strategies exhibit limited anti-tumor activity, when they are used alone. In this study, we explore whether combination of cytokine-induced killer (CIK) adoptive immunotherapy with oncolytic adenovirus-mediated transfer of human interleukin-12 (hIL-12) gene induce the enhanced antitumor potency. Our results showed that oncolytic adenovirus carrying hIL-12 (AdCN205-IL12) could produce high levels of hIL-12 in liver cancer cells, as compared with replication-defective adenovirus expressing hIL-12 (Ad-IL12). AdCN205-IL12 could specifically induce cytotoxocity to liver cancer cells. Combination of CIK cells with AdCN205-IL12 could induce higher antitumor activity to liver cancer cells *in vitro* than that induced by either CIK or AdCN205-IL12 alone, or combination of CIK and control vector AdCN205-GFP. Furthermore, treatment of the established liver tumors with the combined therapy of CIK cells and AdCN205-IL12 resulted in tumor regression and long-term survival. High level expression of hIL-12 in tumor tissues could increase traffic of CIK cells to tumor tissues and enhance their antitumor activities. Our study provides a novel strategy for the therapy of cancer by the combination of CIK adoptive immunotherapy with oncolytic adenovirus-mediated transfer of immune stimulatory molecule hIL-12.

## Introduction

As malignant diseases, liver cancer still holds a very high mortality rate despite of progression in the cutting edge medical technology. Recently, the application of cell-based immunotherapy for the treatment of malignant tumors has achieved encouraging results. Various types of immune cells have been used, including dendritic cells, lymphokine activated killer cells, natural killer cells, cytotoxic T cells, and cytokine-induced killer (CIK) cells [1], [2], [3], [4], [5]. Among them, *ex vivo* expanded CIK cells with both NK and T cell properties exhibit the most therapeutic effect in different experimental tumor models [6], [7], [8]. These cells are generated from peripheral blood mononuclear cells (PBMCs) by the sequential addition of interferon-γ (IFN-γ), anti-CD3 antibody, interleukin-1α and interleukin-2, and represented as heterogeneous cell populations including CD3+CD56+ cells with high antitumor activity [6]. Clinical studies indicated that therapy with CIK cells alone exhibited minor response in patients with high tumor burthen [9]. But as an adjuvant immunotherapy, CIK cells might prevent recurrence and improve quality of life and progression-free survival rates [9], [10].

To improve the therapeutic effect, the combined treatment strategy was suggested future direction [11], [12], [13]. Gene therapy has emerged as a powerful tool to regulate biological functions in diseased tissues and to treat cancers [14], [15]. Oncolytic viruses not only have capacity to express therapeutic genes in tumor cells but also can be used as a direct tumor-destruction medicament. For safety, oncolytic viral replication must be controlled strictly within tumor cells. Thus, the different types of viruses have been genetically modified, including vaccinia, adenovirus, herpes simplex virus type I, reovirus and Newcastle disease virus [16], [17], [18], [19], [20]. One of the common strategies used to design oncolytic adenoviruses is to modify adenoviral E1A protein. The CR2 region of adenoviral E1A binds to retinoblastoma protein (RB). and the RB-related proteins which regulate the E2F family of transcription factors, and induces quiescent cells to enter S-phase. Since the tumor cells often have dysfunctional RB, and uncontrolled cell cycle, deletion of CR2 region allows this engineered adenovirus to selectively replicate in tumor cells but not in quiescent normal cells [21], [22]. We have constructed several conditionally replicative adenovirus systems which viral replication was only occurred in cancer cells with high expression of hTERT and abnormal cell cycle checkpoint [22], [23]. However, among these oncolytic adenoviruses, therapeutic genes were controlled by exogenous constitutive promoters. Thus, expression of therapeutic genes in normal tissue may induce undesired effect even if the virus does not replicate [24]. To overcome this limitation, we have developed the AdCN205 system which therapeutic gene expression is controlled by adenovirus E3 endogenous promoter. We have proven that this vector could express therapeutic gene in a predictable and safe manner [25].

Cytokines were reported to enhance CIK proliferation and antitumor efficacy in *ex vivo* culture or *in vivo* combined administration [5], [26], [27]. Our previous studies have indicated that interleukine-12 (IL-12) based gene therapy exerted strong antitumor activity in preclinical tumor models and human clinical trials [28], [29], [30]. Recently, Helms *el at.* showed that combination of CIK cells with IL-12 immunotherapy resulted in increased efficacy in a preclinical breast cancer model [26]. In the current study, we explore whether the enhanced antitumor activity can be achieved by the combination of adoptive immunotherapy of CIK cells with oncolytic adenovirus expressing hIL-12 (AdCN205-IL12). Our data indicate that combined therapy with CIK cells and oncolytic adenovirus expressing hIL-12 can induce the enhanced antitumor activity.

## Results

### 1. The Construction of AdCN205-IL12 Virus

**Figure 1:**
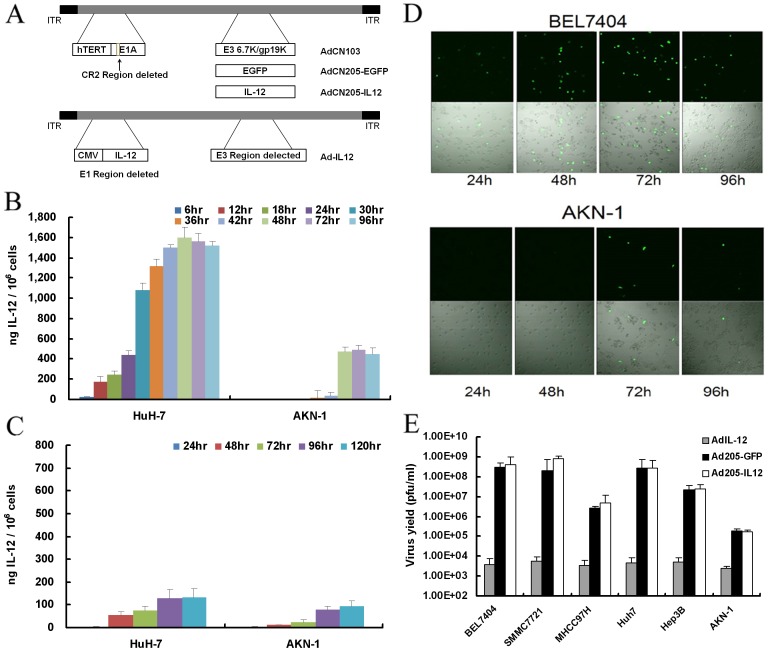
Construction and characterization of AdCN205-IL12. (**A**) Schematic description of the structures of AdCN103, AdCN205-GFP and AdCN205-IL12. In AdCN103, the E1A promoter was replaced by hTERT promoter and deletion of the adenoviral genome 923 to 946 nucleotides, which enables viral replication within malignant cells with abnormal RB functions. In AdCN205-GFP and AdCN205-IL12, E3 6.7 K/gp19K genes were substituted by GFP reporter gene and hIL-12 therapeutic gene, respectively. (**B**) Expression of transgene in the cells after infection with oncolytic adenoviruses. Tumor cells (HuH-7) and normal cells (AKN-1) were infected with AdCN205-IL12 at the multiplicity of infection (MOI) of 10. At different time points, the culture supernatant was collected for determination of hIL-12 levels by ELISA. (**C**) hIL-12 level in the cells infected with Ad-IL12 at the MOI of 10. The data was presented as the mean ± SD of three independent experiments. (**D**) Representative photomicrographs were obtained from BEL7404 and AKN-1 infected with AdCN205-EGFP at the MOI of 10. Original magnification, 200×. (**E**) Selective replication of oncolytic adenoviral vectors *in vitro*. Tumor cells (BEL7404, SMMC7721, MHCC-97H, HuH-7, Hep3B) and normal cells (AKN-1) were infected with AdCN205-GFP, AdCN205-IL12 or Ad-IL12 at the MOI of 10 respectively. At 48 hours after viral infection, cells and medium were harvested, and viral particles were released by three cycles of freezing and thawing. The viral titers were measured by using plaque assay of QuickTiter™ Adenovirus Titer Immunoassay Kit on HEK293 cells. The data was presented as the mean ± SD of three independent experiments.

Previously, we developed a double-controlled oncolytic adenovirus system, AdCN205, in which hTERT promoter was used to control the expression of CR2 deleted E1A region and the 6.7 K/gp19K of E3 region were substituted by the exogenous genes [25]. This vector allows selective adenoviral replication in the tumor cells harboring overexpression of hTERT and dysfunction of RB. The exogenous genes in the vector controlled by the adenovirus endogenous E3 promoter are expressed in the tumor cells following virus replication. In the present study, we constructed AdCN205-IL12 by replacing GFP with hIL12 gene. The structures of AdCN205-GFP, AdCN205-IL12 and Ad-IL12 were shown in **Figure 1** **A**.

### 2. Selective Replication and Gene Expression of Oncolytic Adenoviral Vectors *in vitro*

To show kinetics of hIL-12 expression in the cells after infection with AdCN205-IL12, we infected tumor (HuH-7) and normal (AKN-1) cells with AdCN205-IL12. Our data showed that hIL-12 was detectable at 6 h in tumor cells after infection. There was a steady increase of hIL-12 expression up to 48 hours, which was maintained at high level until 96 hours after infection (**Figure 1B**). In contrast, hIL-12 was undetectable in normal cells until 30 hours after infection and we could only detect extremely low level of hIL-12 after that (**Figure 1B**). However, similar kinetics and level of hIL-12 expression were found in both tumor and normal cells infected with replication defective adenoviral vector Ad-IL12, in which hIL-12 was controlled by cytomegalovirus promoter (**Figure 1C**). The expression level of hIL-12 was about 12 times higher in the tumor cells infected with AdCN205-IL12 than that infected with Ad-IL12, indicating that oncolytic adenoviral vector could efficiency expression transgene. To confirm the specific expression of transgene in cancer cells by our vector system, we further infected both cancer and normal cells with control vector AdCN205-GFP. As shown in the **Figure 1D**, the high level of GFP expression was only observed in tumor cells accompanied with cytopathic effect, and no or low GFP expression was observed in normal cells after infection with AdCN205-GFP. Furthermore, we found that both of AdCN205-IL12 and AdCN205-GFP could specifically replicate in tumor cells but not in normal cells (**Figure 1E**). These data indicated that the difference in the kinetics and level of transgene expression may be due to tumor specific viral replication induced by oncolytic adenoviral vectors.

### 3. Characteristics of CIK Cell Populations

Results from phenotypic analysis of the cultured CIK by flow cytometry were presented in **Figure S1**. After 14-days culture, the percentage of CD3/CD8 double-positive, CD3/CD4 double-positive, CD3/CD56 double-positive, CD4/CD25 double-positive, and NKG2D positive cells was 69.2±4.75%, 12.13±1.58%, 25.48±4.77%, 3.38±0.83%, 83.5±3.87% in CIK cultures, separately. These data is consistent with previous report [27]. To test whether adenovirus type 5 could infect CIK cells, we infected CIK cells with Ad5-GFP virus at different MOI (1–100), which GFP gene expression were controlled by CMV promoter. After 48 hours, GFP expression was analyzed by flow cytometry. Results showed that no GFP expression was observed even at high MOI. However, chimeric adenovirus vector Ad5/11-GFP [31] could efficiently transduce CIK cells (**Figure S2**).

### 4. Cytotoxic Effects of CIK Cell and Oncolytic Adenoviral Vectors on Liver Tumor Cells *in vitro*

**Figure 2:**
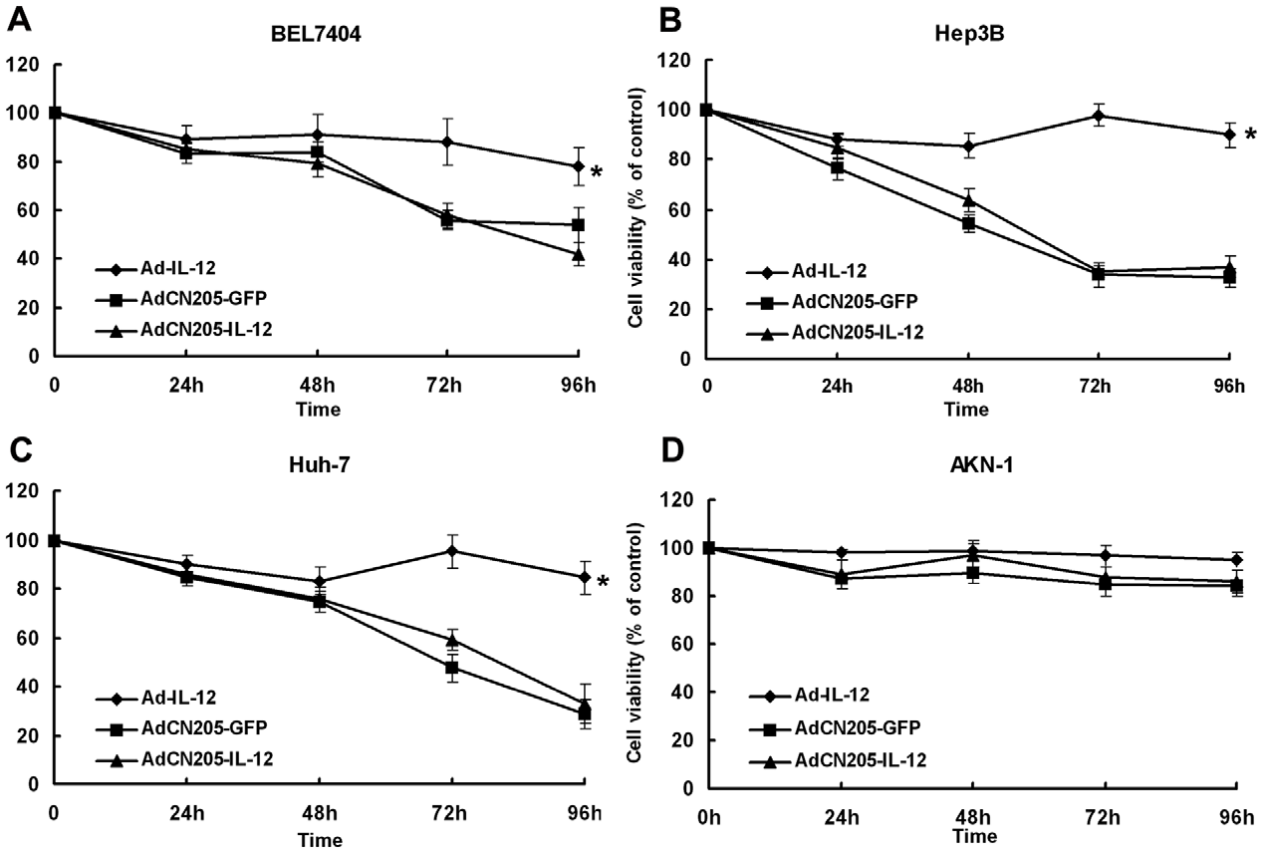
The cytotoxcity effect of oncolytic adenoviruses *in vitro*. Liver tumor cells (BEL7404, Hep3B and HuH-7) and normal cells (AKN-1) were infected with AdCN205-GFP, AdCN205-IL12 or Ad-IL12, at the MOI of 10. At different time points after infection, the cell survival was measured by CCK8 assay. Results were expressed as percentage of untreated control. The data was presented as the mean ± SD of three independent experiments. *p* = 0.035357 (AdCN205-GFP) and 0.033118 (AdCN205-IL12) compared with Ad-IL12 in figure A; P = 0.02159 (AdCN205-GFP) and 0.042144 (AdCN205-IL12) compared with Ad-IL12 in figure B; P = 0.045401 (AdCN205-GFP) and 0.048508 (AdCN205-IL12) compared with Ad-IL12 in figure C.

To investigate the antitumor ability of the oncolytic adenovirus with therapeutic gene IL-12, we performed cytotoxicity assay after infection with adenoviruses. As shown in **Figure 2**, replication-defective adenovirus Ad-IL12 did not induce cytotoxicity, whereas AdCN205-GFP or AdCN205-IL12 induced significant cytotoxicity to the tumor cells. The cytotoxic effect on the tumor cells had no obvious difference between AdCN205-GFP and AdCN205-IL12. In contrast, AdCN205-GFP, AdCN205-IL12, and Ad-IL12 did not induce any cytotoxicity to the normal cells. These results indicate that AdCN205-GFP and AdCN205-IL12 can selectively replicate in liver cancer cells to kill them and IL-12 gene does not exert direct biological effect on cancer cells *in vitro*.

**Figure 3:**
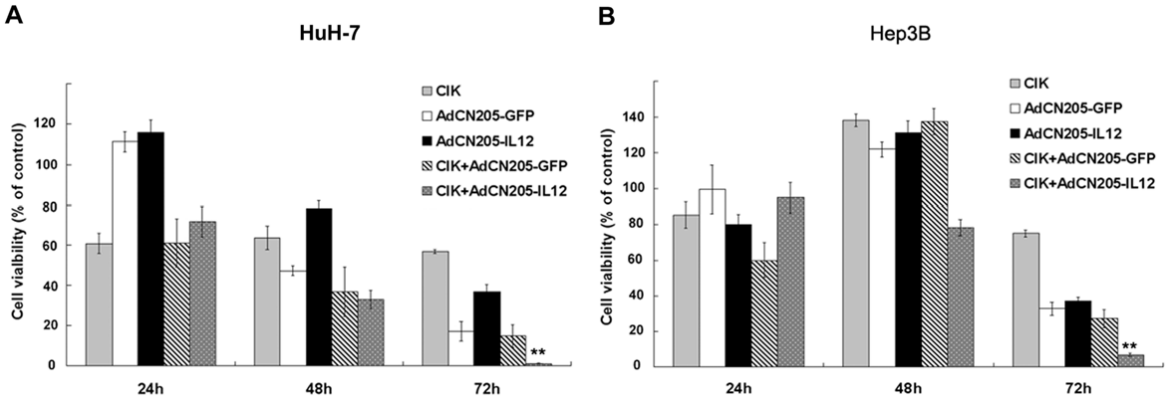
The cytotoxcity effect of combination of CIK with oncolytic adenoviruses *in vitro*. Liver tumor cells (HuH-7 and Hep3B) were treated with CIK cells alone at effector:target ratio of 10∶1, or AdCN205-GFP, AdCN205-IL12 at the MOI of 10, or treated with both CIK at effector:target ratio of 10∶1 and AdCN205-GFP or AdCN205-IL12 at the MOI of 10. The cell survival was determined by measuring luciferase activity. Results were expressed as percentage of untreated control. The data was presented as the mean ± SD of three independent experiments.

To investigate the cytotoxicity of CIK and CIK combined with oncolytic adenoviral vectors on cancer cells, cytotoxocity assays were performed by treatment of cancer cells (HuH-7 and Hep3B) with CIK cells alone or with oncolytic adenoviral vectors. Since the presence of CIK cells in the assay may cause interference in conventional cell viability assay, we engineered cancer cells expressing luciferase reporter gene to monitor survival of cancer cells by measurement of luminescence intensity from the survived cancer cells. Our results showed that CIK cells could exert minor cytotoxic effect on the cancer cells when effector:target ratio was 10∶1. AdCN205-GFP and AdCN205-IL12 induced cytotoxicity at time-dependent manner. The combination of CIK cells with oncolytic adenovirus showed stronger cytotoxocity than either of them used alone. Particularly, combination of CIK with AdCN205-IL12 could induce significant cytotoxicity to the cancer cells, as compared with combination of CIK cells with AdCN205-GFP (**Figure 3**).

### 5. Antitumor Activity of the Combined Therapy in the Animal Model with Established Tumor

**Figure 4:**
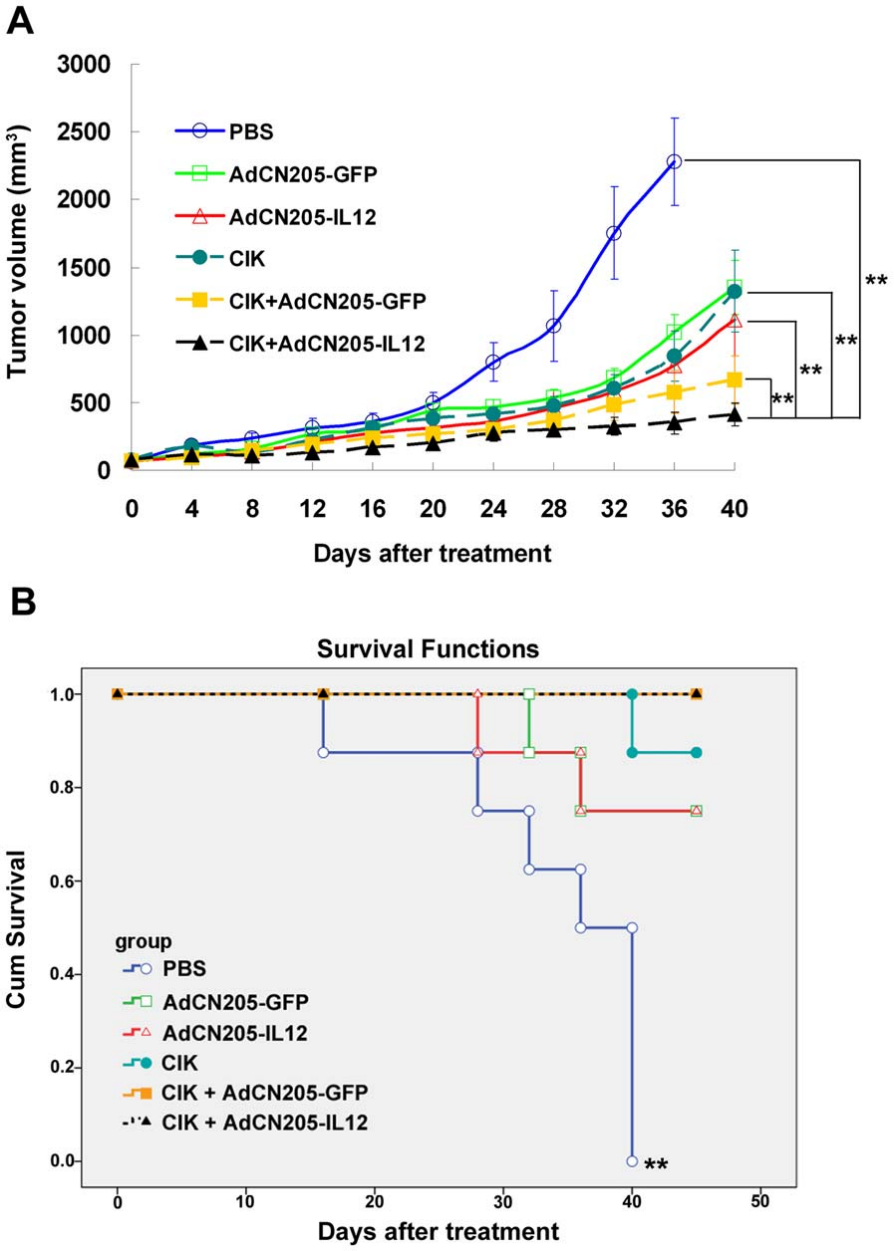
Antitumor activity in the established tumor in an animal model. Tumors were established by injection of HuH-7 cells s.c. into the right flank of SCID mice. The tumor-bearing animals were treated with either 2×10^8^ ifu of different vectors by intratumoral injection or 1×10^7^ CIK by intravenous injection, or combination of both treatment. PBS was used as control group. (**A**) The size of tumor was measured every 4 days and tumor volume was calculated. The data was presented as the mean ± SEM (n = 8). The result of student *t* test shown CIK plus AdCN205-IL12 group have extremely significant difference with PBS group (*p* = 0.0062), AdCN205-GFP group (*p* = 0.0058), AdCN205-IL12 group(*p* = 0.0098), CIK group (*p* = 0.0081), and CIK plus AdCN205-GFP group (*p* = 0.0052) (**B**) Kaplan-Meier survival. The result of log-rank statistical test shown PBS group have extremely significant difference with AdCN205-GFP group (*p* = 0.009), AdCN205-IL12 group(*p* = 0.01), CIK group (*p*<0.0001), CIK plus AdCN205-GFP group (*p*<0.0001) and CIK plus AdCN205-Il12 group (*p*<0.0001). The other group have no significant different with each other group.

To investigate the antitumor potency of CIK cells combined with oncolytic adenoviral vectors *in vivo*, we treated the established liver tumors (HuH-7) in the SCID mouse model by intratumoral injection of AdCN205-GFP, AdCN205-IL12 or PBS and intravenous injection of CIK cells or PBS. Our data showed that animals receiving PBS expressed progressive tumor growth. Treatment with AdCN205-GFP, AdCN205-IL12, and CIK resulted in minor inhibition of tumor growth (Figure 4A). The combined CIK and AdCN205-GFP exhibited stronger antitumor activity than either of them used alone. In contrast, the combined treatment with CIK and AdCN205-IL12 significantly inhibited tumor growth, as compared with the combination of CIK with AdCN205-GFP (*p* = 0.0052; Figure 4A). Two of eight animals treated with CIK and AdCN205-IL12 had complete tumor regression, and this combined therapy resulted in long-term survival (Figure 4B). The log-rank statistical test showed that PBS group had significant difference with AdCN205-GFP group (*p* = 0.009), AdCN205-IL12 group(*p* = 0.01), CIK group (*p*<0.0001), CIK plus AdCN205-GFP group (*p*<0.0001) and CIK plus AdCN205-Il12 group (*p*<0.0001). The other groups had no significant difference among groups.

**Figure 5:**
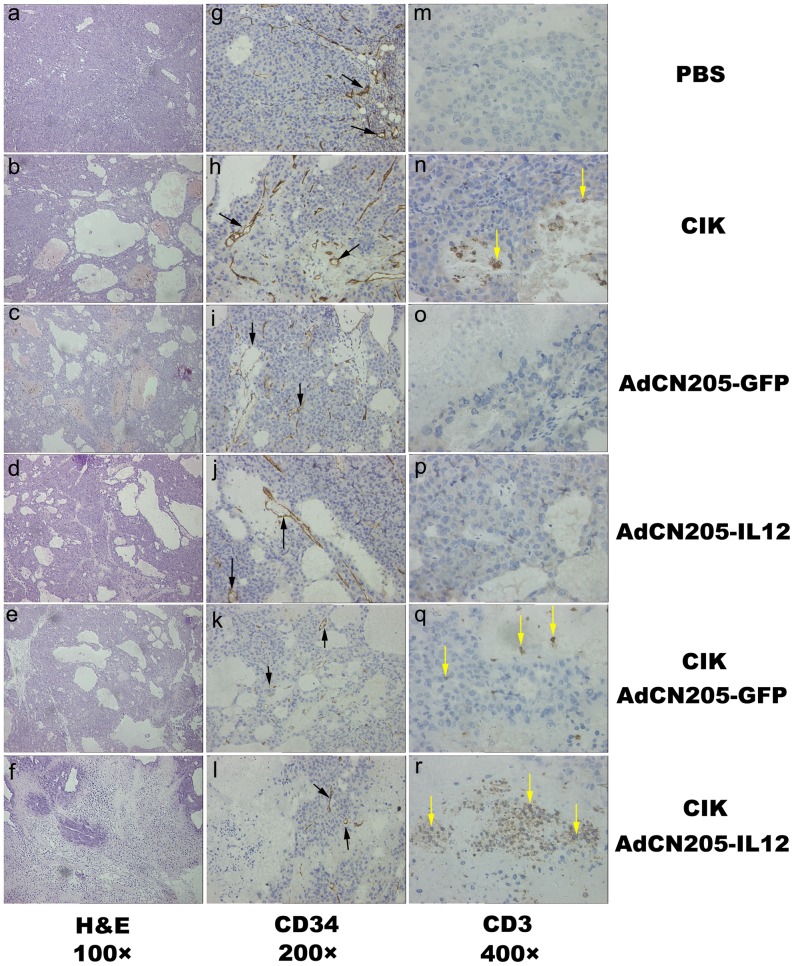
Histologic analysis of tumor sections from the mice that were treated with PBS (a, g, and m); CIK (b, h and n); AdCN205-GFP (c, i and o); AdCN205-IL12 (d, j and p); CIK combined with AdCN205-GFP (e, k and q) or CIK combined with AdCN205-IL12 (f, l and r). a to f, H&E staining of tumor sections, showing the areas of necrosis in tumors. Original magnification, 100×. g to l, Immunohistochemical analysis of tumor blood vessel in tumor sections using anti-mouse CD34 antibody staining (black arrow). Original magnification, 200×. m to r, Immunohistochemical analysis of human CIK cells in tumor sections using anti-human CD3 antibody staining (yellow arrow). Original magnification, 400×.

Immunohistochemical detection of blood vessels in the tumor sections by anti-mouse CD34 antibody revealed that presented a mass of blood vessels in PBS or CIK group, whereas only few blood vessels which detected in AdCN205-GFP, AdCN205-IL12, CIK plus AdCN205-GFP or AdCN205-IL12 group (**Figure 5**). Further quantification showed that the vascular density of tumors treated with CIK plus AdCN205-GFP or AdCN205-IL12 were significantly lower than tumors treated with PBS and CIK cells (**Figure S3).** Furthermore, we detected human CIK cells in tumors by immunohistochemical analysis using anti-human CD3 antibody. Our result showed that infiltration of human CIK was largely enhanced in the tumors treated with CIK combined with AdCN205-IL12 (**Figure 5**).

### 6. Therapeutic Gene Expression in the Tumors After Treatment

**Figure 6:**
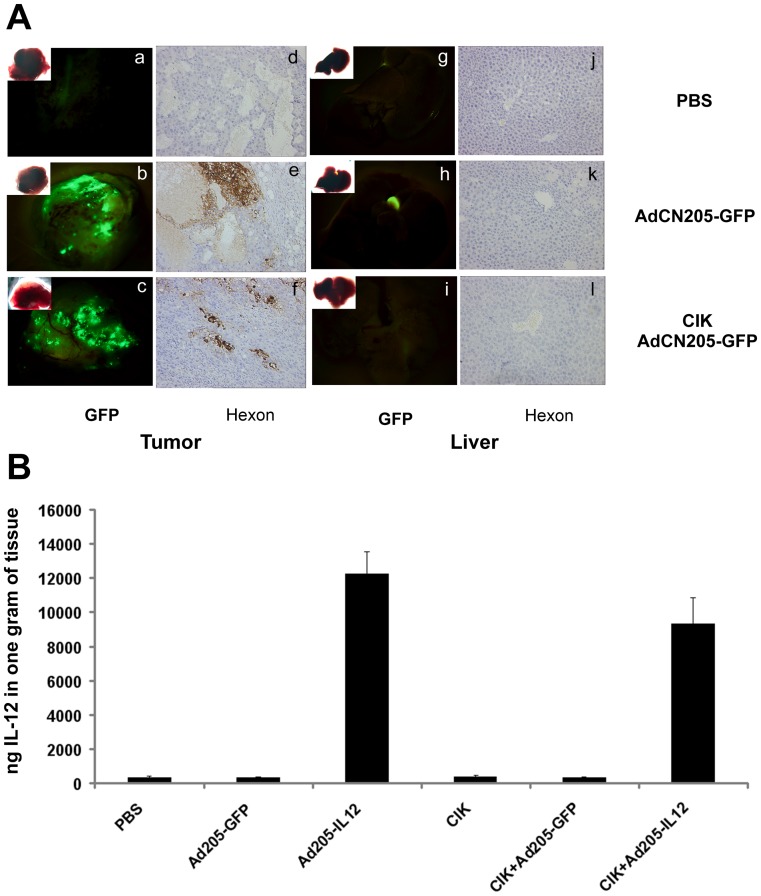
The expression of exogenous gene in tumor sections and liver tissues. (A). Histologic and fluorescence analysis of tumor and liver sections from mice treated with PBS (a, d, g and j), AdCN205-GFP (b, e, h and k), CIK plus AdCN205-GFP (c, f, i and l). a to c, GFP was observed by fluorescence microscopy in the tumor tissue treated with PBS, AdCN205-GFP, or CIK plus AdCN205-GFP. d to f, Immunohistochemical staining for adenoviral hexon protein on the tumor sections (brown staining). g to i, GFP was observed by fluorescence microscopy in liver tissues. j to l, Immunohistochemical staining for adenoviral hexon protein on the liver sections. Original magnification, 200×. (B). The expression of hIL-12 in tumor fraction. Tumor tissues collected at the day of 6 after treatment. The hIL-12 was detected by ELISA. The data was presented as the mean ± SD (n = 3 per group).

To investigate whether exogenous therapeutic gene could be expressed in the tumors *in vivo*, mice were sacrificed after 6 days of treatment. We found that GFP was highly expressed in the tumor sections from animals treated with AdCN205-GFP and CIK plus AdCN205-GFP, whereas expression of GFP was undetectable in liver tissues, which is the primary infected tissue for adenovirus (**Figure 6A**). Similarly, the expression of hIL-12 was detected only in the tumors from the animals treated with AdCN205-IL12 and CIK plus AdCN205-IL12, while the combined treatment of CIK and oncolytic adenoviral vectors did not reduce the expression of transgenes (**Figure 6B**). Immunohistochemistry analysis showed that adenovirus hexon protein could only be detected in the tumor but not in liver sections when treated with AdCN205-GFP, AdCN205-IL12 plus CIK or not (**Figure 6A**). Thus, treatment with CIK cells does not affect the specificity of oncolytic adenovirus replication.

## Discussion

Adaptive immunotherapy with CIK cells has been proven to be effective and safe for the treatment of cancers in the preclinical and clinical studies [9], [32]. It has been shown that therapy with CIK cells exerts antitumor activity with high efficiency in the prevention of tumor recurrence after surgical removal of primary tumors [9], [33]. However, in the late stage of cancer patients, response rate is low and the tumor volume reduction is hardly achieved, due to the high tumor burden and the presence of immunosuppressive environment [9]. To improve the curative effect, combined therapeutic strategies are recommended [26], [34], [35], [36]. Thus, exploring a novel strategy for decreasing tumor volume and improving tumor immunity may benefit for the following CIK adaptive immunotherapy.

Gene therapy offers a new approach to treat cancer. Oncolytic virus-mediated gene therapy combines the advantages of both gene therapy and virotherapy, which can not only deliver the therapeutic gene into the tumor tissues, but also directly reduce the tumor burden by oncolytic effect [23], [25]. After the oncolytic effect, the release of tumor antigens may provide an opportunity for antigen-presenting by APC cells to enhance the tumor immune response [37], [38], [39]. However, immunity against cancer is often limited by weak immunogenicity of tumor antigens. Thus, transfer of genes encoding immune stimulatory cytokines has been used with remarkable success to suppress cancer and enhance immunity both in the animal models and the clinical trials [30]. Among immune stimulatory cytokines, IL-12 exerts the most potent antitumor activity by inducing a Th l type of response, and activating NK and CTL cells. IL-12 also inhibits tumor neo-angiogenesis [40], [41] and enhances the expression of adhesion molecules on the endothelial cells, thereby facilitating the traffic of lymphocytes to the tumor. Recently, Helms *el at.* reported that IL-12 could improve recruitment of CIK cells and the increased antitumor activity could be achieved by combining IL-12 cytokine therapy with CIK based adoptive immunotherapy [26].

In this study, we explored a new strategy for the therapy of liver cancer by combination of oncolytic adenovirus expressing hIL-12 with CIK adaptive immunotherapy. The basis of this strategy is that viral replication kills tumor cells and expresses hIL-12. Meanwhile hIL-12 strengthens antitumor activity by increasing traffic of CIK cell to tumor regions and cytotoxic effect of CIK cells. For this purpose, we firstly constructed oncolytic adenovirus expressing hIL-12. As expected, high level of hIL-12 could be expressed in the cancer cells and only low level of hIL-12 could be detected in the normal cells after infection with oncolytic adenovirus carrying hIL-12 gene. In contrast, our previous replication defective adenovirus construct carrying IL-12 gene [30] only expressed low level of IL-12 both in the cancer cells and normal cells. Thus, the oncolytic adenovirus expressing IL-12 can serve a better delivery system for cancer therapy. We further demonstrated that both AdCN205-GFP and AdCN205-IL12 could specifically replicate and induce comparable cytotoxicity in liver cancer cells, indicating that hIL-12 has no direct effect on cancer cells survival. However, if AdCN205-IL12 was combined with CIK cells, the enhanced antitumor effect was observed.

Furthermore, this dramatic antitumor activity was also observed in an animal model with the established liver tumors. Complete tumor remission was observed in two of eight mice treated with the combination of CIK with AdCN205-IL12. And the long term survival was achieved by this therapy. The efficacy of combined treatment was superior to other tested treatment regimens including AdCN205-IL12 and CIK administrated separately. Although the tumor remission was not observed in the tumor-bearing animals treated with CIK combined with AdCN205-GFP, tumor suppression and animal survival was significantly improved, as compared with CIK or AdCN205-GFP treatment alone, implying that the oncolytic virus therapy and CIK immunotherapy have enhanced effects, and therapeutic gene IL-12 can further amplify these effects.

Our previous preclinical and clinical cancer gene therapy studies using hIL-12 indicated that hIL-12 could induce antitumor activity by reducing tumor angiogenesis and increasing lymphocyte traffic to tumor tissue overexpressing adhesion molecules [30]. In this study, we found that tumors treated with AdCN205-IL12 alone or combined with CIK resulted in obvious decrease in the blood vessel destiny. In addition, the increased lymphocyte infiltration was found in the tumor-bearing animals after treatment with AdCN205-IL12 and CIK cells, indicating that high level of hIL-12 expression in the tumor tissue can enhance traffic of CIK cells to tumor tissue and increase antitumor activity of CIK cells.

Although IL-12 has been reported to have the lethal toxicity by intravenous administration in a phase II clinical trial [42], [43], no obvious side effect was observed in the clinical application of IL-12 expressing adenovirus vector by intratumor injection [30]. For the avoidance of IL-12 gene expression in the non-tumor tissue, we employed the AdCN205 system vector to allow hIL-12 expression only in those tumor cells with high level hTERT expression and dysfunctional RB [25]. Thus, this system further guarantees safety of using IL-12 for cancer therapy.

In summary, we provide a novel strategy for cancer treatment by combination of CIK adoptive immunotherapy with oncolytic adenovirus expressing immune stimulatory molecule hIL-12.

## Materials and Methods

### 1. Ethics Statement

We didn’t use of any non-human primates in the research. The SCID mice in this research were obtained from the Third Military Medical University and maintained at pathogen-free conditions. All procedures were done according to protocols approved by the Institutional Review Board of the Southwest Hospital, Third Military Medical University and conformed to the NIH guidelines on the ethical use of animals.

### 2. Generation of CIK Cells

Human peripheral blood mononuclear cells (PBMCs) were obtained from health donors with written informed consent from all persons according to protocols approved by the Institutional Review Board of the Southwest Hospital, Third Military Medical University. CIK cells were generated as described previously [27]. Briefly, human PBMCs were cultured in RPMI 1640 medium (Gibco, Inc) containing 10% fetal calf serum (Gibco, Inc), 25 Mm HEPES, 100 U/ml penicillin and 100 *µ*g/ml streptomycin. Human recombinant IFN-γ (Peprotech, Inc) were added at 1000 U/ml on day 0. After 24 hours of incubation, 50 ng/ml of antibody against CD3 (eBioscience, Inc), 100 U/ml IL-1α (Invitrogen, Inc) and 300 U/ml IL-2 (Peprotech, Inc) were added. Cells were incubated at 37°C in a humidified atmosphere of 5% CO_2_ and subcultured every 3 days in fresh complete medium and 300 U/ml IL-2 at 3×10^6^ cells/ml. The cells were cultured until 14 days to obtain CIK cells.

### 3. Cell Lines and Cell Culture

Human hepatocellular carcinoma (HCC) cell lines Hep3B (HBs ^pos^_,_ undetected Rb), were purchased from American Tissue Culture Collection (ATCC, Manassas, VA, USA), BEL7404 (HBs^neg^, Rb^wt^), SMMC7721 (HBs^neg^, Rb^wt^), MHCC97H (HBs^pos^, Rb^wt^ hyperphosphorylated) and HuH-7 (HBs^neg^, Rb^wt^ hyperphosphorylated) [44], [45], [46] were purchased from the Shanghai Cell Collection, China. Human normal liver cell line AKN-1 (HBs^neg^, Rb^wt^) was gifted by Pro. Stephen C. Strom [47]. HEK293 were obtained from Microbix Biosystems Inc (Etobicoke, ON, Canada). Cells were cultured in Dulbecco’s modified essential medium (DMEM) supplemented with 10% heat-inactivated fetal bovine serum (FBS) (Gibico, Inc), 4 mM glutamine, 50 U/ml penicillin, and 50 *µ*g/ml streptomycin at 37°C in a humidified atmosphere with 5% CO_2_. The expression level hTERT in HCC and normal cell lines was measured by qRT-PCR. Our result showed that HCC cell lines expressed higher level of hTERT than normal liver cell line (**Figure S4**).

### 4. Virus Construction

Human hIL12-p40-Δp35 cDNA was obtained by PCR from the Ad-IL12 vector described before [30]. The amplification for hIL12-p40 was done with forward (5′-CCATGGGTCACCAGCAGTTG-3′) and reverse (5′-CTCGAGCCGCCGCCGCCGCCGCCACTGCAGGGCACAGAT-3′) primers. The amplification for hIL12-Δp35 was done with forward (5′-CCATGGCATGCTCGAGAAACCTCCCCGT-3′) and reverse (5′-GGTACCTTAGGAAGCATTCAGAT-3′) primers. The PCR fragments were cloned to pMD-18-T-hIL12-p40-Δp35 after digested by *Xho*I. hIL12-p40-Δp35 gene was excised from pMD-18-T-hIL12-p40-Δp35 with *Nco*I and *Kpn*I and clone to pCN204 plasmid. The construction of pCN205-hIL12-p40-Δp35 was generated according to the standard protocol described before [25]. Homologous recombination between pCN204-hIL12-p40-Δp35 and pCN103 plasmids carrying oncolytic adenoviral backbone was done in *E.coli* strain, BJ5183 to create pCN205- hIL12-p40-Δp35 (pCN205-IL12). Viral particles were produced in HEK293 cells by transfection with *Pac*I-digested pCN205-GFP and pCN205-IL12 to obtain recombinant AdCN205-GFP and AdCN205-IL12. All viruses were propagated and purified by CsCl gradient centrifugation using standard methods. Functional titer (infectious units/ml) was determined on HEK293 cells according to QuickTiter Adenovirus Titer Immunoassay Kit (Cell Biolabs, Inc).

### 5. RNA Purification and Reverse Transcription PCR

Total RNA was purified from cultured cells with the RNAiso Plus reagent (TaKaRa, Shiga, Japan), and 2 µg of total RNA were reverse-transcribed with PrimeScript RT reagent Kit (TaKaRa, Shiga, Japan). 2 µl of the diluted RT reaction product was added in the SYBR Premix Ex Taq II (TaKaRa, Shiga, Japan) RT-PCR reaction mixture, and detected by the CFX96™ Real-Time PCR Detection System (Bio-Rad, Inc). The PCR primers for hTERT are forward (5′GGCTGTGCCACCAAGCATTC 3′) and reverse (5′AGGGCTGCTGGTGTCTGCTC3′), for GAPDH are forward (5′TTGCAGTGGCAAAGTGGAGA3′) and reverse (5′CGTGGTTCACACCCATCACAA3′).

### 6. *In vitro* Transduction Studies

For adherent cells, the day before infection, 3×10^5^ cells per well (24-well plate) were seeded. The next day, attached cells were counted and viruses were added at the multiplicities of infection (MOI) indicated in the figure legends in 1 ml of growth medium. Cells were incubated with virus for 6 hours, then washed twice with PBS and incubated at 37°C. Percentages of GFP-positive cells were detected by fluorescent microscopy. Cells growing in suspension were washed once with phosphate-buffered saline (PBS) by centrifuging at 170×*g* for 5 minutes at 4°C before infection and resuspended in growth medium at a concentration of 3×10^6^ cells/ml in serum-free medium. Virus at the MOI indicated in the figure legends was added to a total volume of 200 µl and incubated for 6 hours at 37°C in a humidified atmosphere with 5% CO_2_. Cells were washed with PBS by centrifuging at 170×*g* for 5 minutes at 4°C, and cultured in completed medium for 48 hours at 37°C in a humidified atmosphere with 5% CO_2_. Percentages of GFP-positive cells and mean fluorescence were determined by flow cytometry.

### 7. Viral Progeny Assay

To determine viral replication capacity, tumor or normal cells were infected with Ad-IL12, AdCN205-GFP, and AdCN205-IL12 at the multiplicity of infection of 10. Adenoviruses were removed after 6 hours of infection. The cells were washed twice with PBS and cultured in the completed medium at 37°C. After 48 hours, the cells were collected by trypsinization and centrifugation and then lysed with three cycles of freeze thawing. After centrifugation, the supernatant was collected. Viral titers were determined by adenovirus Titer Immunoassay Kit on HEK293 cells.

### 8. Cytotoxicity Assay

Cells were plated in 96-well plates and treated with various adenoviruses. At the indicated times, medium was removed and changed to fresh medium containing 10 µl CCK-8 (Cell counting kit-8, Dojindo). Cells were incubated at 37°C for 2 hours. Absorbance was read on a spectral Scanning Multimode Reader (Varioskan Flash,Thermo Scientific) at 450 nm. To determine the CIK-mediated cellular cytotoxicity, tumor cell lysis by effector cells was assessed by measurement of the luciferase activity of surviving target cells. Target cells expressing luciferase were plated into black-walled 96-well plates at 1×10^4^ cells/well. Effecter cells were then added at effecter-to-target ratios 10 to 1; wells representing all ratios of cells and target cells only wells were plated in triplicate and incubated for 24 to 72 hours at 37°C, 5%CO_2_. The Luciferase Assay Reagent was then added to each well (Promega inc) and light output measured on luminometric spectral reader (Varioskan Flash, Thermo Scientific). Percent cytotoxicity was then determined relative to control wells.

### 9. Immunohistochemistry, Fluorescence, and Flow Cytometry Studies

Immunohistochemistry: Deparaffinaged tumor sections were treated with rabbit anti-human CD3 polyclonal antibody (1∶50; Abcam), rabbit anti-human CD34 monoclonal antibody (1∶200; Abcam), goat anti Adenovirus Hexon polyclonal (diluted 1∶500; Santa Cruz Biotechnology). After incubation with anti-rabbit or anti-goat secondary antibody, gene expression was detected with 3,3-diaminobenzidine (Sigma) by enhancement with an avidin-biotin reaction ABC kit (Vector Laboratories). The slides were then counterstained with hematoxylin. Fluorescence: The green fluorescence of the AdCN205-GFP infected cells was assayed using a 530-nm/30-nm bandpass filter after illumination with an argon ion laser tuned at 488 nm (AXIO OBSERVER A1, Carl Zeiss, Inc). Tumor sections infected by AdCN205-GFP assayed by a fluorescence stereo microscopy (SZX10, Olympas, Inc). Flow cytometry: Cells were obtained from CIK cultures for phenotype analysis with appropriate monoclonal antibodies, including mouse anti-human CD3-FITC (BD Biosciences, CA, USA), mouse anti-human CD4-PE (BD Biosciences, CA, USA), mouse anti-human CD8-PE-Cy5 (BD Biosciences), mouse anti-human CD25-PE (BD Biosciences, CA, USA), mouse anti-human CD56-PE-Cy5 (BD Biosciences, CA, USA), mouse anti-human CD314-PE (BD Biosciences, CA, USA). 1×10^6^ CIK cells were washed once with PBS containing 1% bovine serum albumin (BSA) and resuspended in 100 µL of PBS/BSA buffer. The cells were incubated with various conjugated monoclonal antibodies for 20 minutes at 4°C, washed twice with PBS, and resuspended in 300 µL of PBS. Flow cytometric analysis was performed on a FACS Arial II flow cytometer (BD Biosciences, CA, USA).

### 10. Enzyme-linked Immunosorbent Assay

The detection of human hIL-12 (P70) in the cell culture supernatant or tissues was performed by using an ELISA kit (R&D Systems) according to the manufacture’s instruction.

### 11. *In vivo* Study of Anti-tumor Effect

Male SCID mice at 3 to 4 weeks of age were obtained from the Third Military Medical University and maintained at pathogen-free conditions. All procedures were done according to institutional guidelines and conformed to the NIH guidelines on the ethical use of animals. HuH-7 cells (1×10^6^) were injected s.c. into the flanks of mice. When the tumors reached to 100 to 150 mm^3^, the animals were randomized into six groups, and treated with either PBS or 2×10^8^ IFU of vectors (AdCN205-GFP or AdCN205-IL12) by intratumoral injection or 1×10^7^ CIK cells by intravenous injection, or intravenous injection 1×10^7^ CIK combined with intratumoral injection of 2×10^8^ IFU vectors (CIK combined with AdCN205-GFP or CIK combined with AdCN205-IL12). Tumors were measured twice weekly and tumor volume was calculated according to the following equation: V (mm^3^) = (width^2^×length)/2. Animal death was documented. Animals were sacrificed and were considered as death, when the diameter of the tumor reached 17 mm or when mean diameter was >15 mm.

### 12. Statistical Analysis

All *in vitro* experiments were performed at least three times. The statistical significance was calculated by analysis of variance when more than two groups were compared or by student *t*-test when only two groups were compared. *p<0.05 and **p<0.01. *In vivo* experiment was performed once with 8 animals in each group. The statistical significance of tumor growth curve was calculated by student *t*-test, and the statistical significance of survival curve was calculated by log-rank statistical test. *p<0.05 and **p<0.01.

## Supporting Information

Figure S1 **The phenotype of CIK cells.** CIK cells were stained with various monoclonal anti-bodies as outlined above. The expression of the CD3, CD56, CD4, CD8, CD16, NKG2D, were coincident with previous described.
(TIF)

Figure S2 **The infection of Ad5-GFP virus in CIK cells.** The GFP positive rate was below 0.2% even in 100 MOI. Chimeric adenovirus vector Ad5/11-GFP used as a positive control. The data was presented as mean ± SD of three independent experiments.
(TIF)

Figure S3 **The vascular density in tumor.** The vascular density was quantified by counting numbers of endothelial cells in 5 random fields per section at 200×magnification. The vascular density from PBS group has significant difference with that from AdCN205-GFP plus CIK (*p* = 0.045499) and AdCN205-IL12 plus CIK group (*p* = 0.035946). The vascular density from CIK group has significant difference with AdCN205-GFP plus CIK (*p* = 0.038171) and AdCN205-IL12 plus CIK group (*p* = 0.029449).
(TIF)

Figure S4 **The detection of hTERT expression in liver cancer and normal liver cell lines by real time PCR.** The expression of hTERT gene of liver cancer cell lines (SMMC7721, BEL7404, MHCC97H, HuH-7 and Hep3B) and normal liver cell (AKN-1) was detected by real time PCR.
(TIF)
