# Supplementary figures and images for: Combined Therapy with Cytokine-Induced Killer Cells and Oncolytic Adenovirus Expressing IL-12 Induce Enhanced Antitumor Activity in Liver Tumor Model
Source: PLoS One. 2012 Sep 18;7(9):e44802. doi: 10.1371/journal.pone.0044802 (PMC3445563; doi:10.1371/journal.pone.0044802)

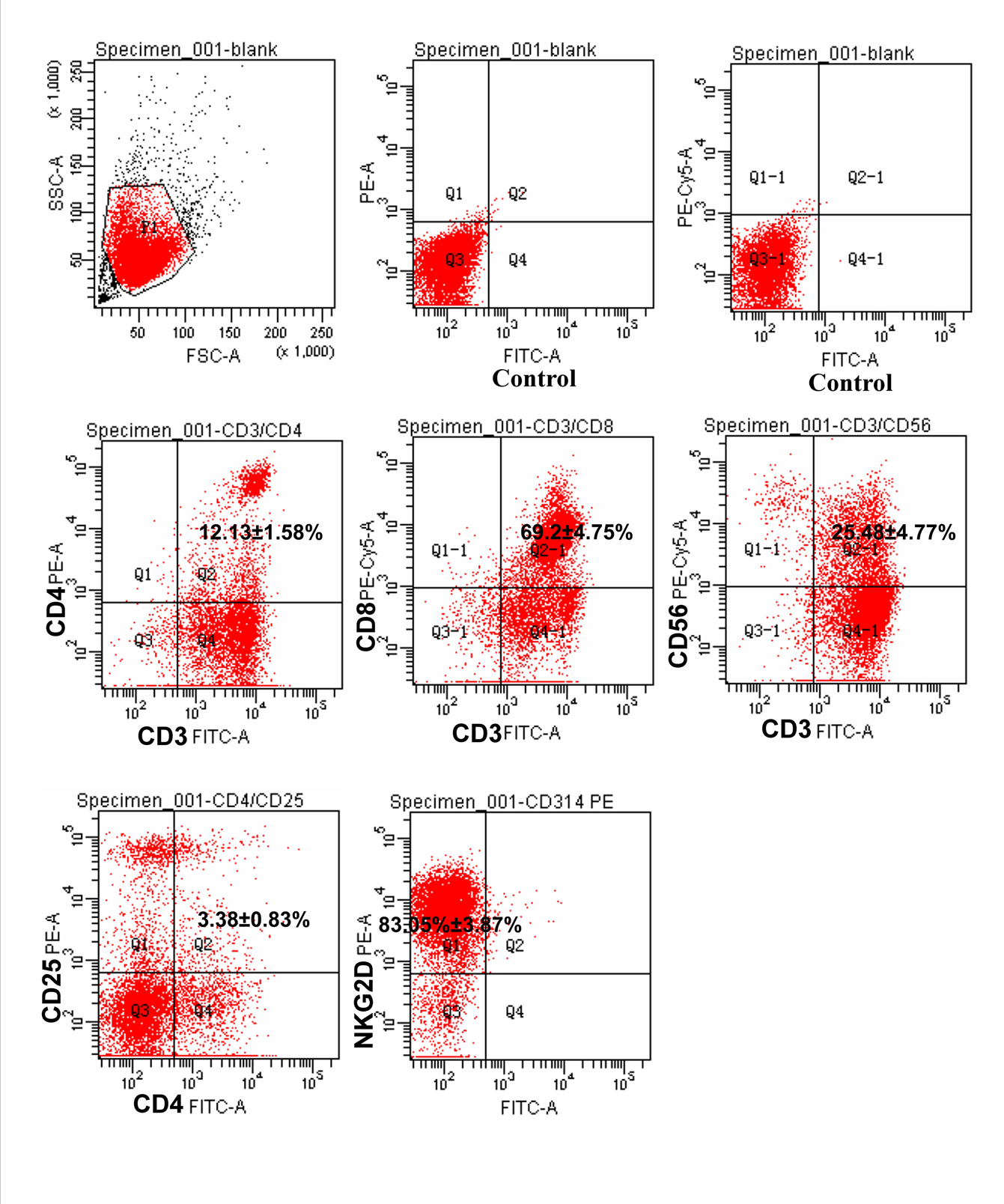

Supplement: Figure S1 — The phenotype of CIK cells. CIK cells were stained with various monoclonal anti-bodies as outlined above. The expression of the CD3, CD56, CD4, CD8, CD16, NKG2D, were coincident with previous described. (TIF) [file pone.0044802.s001.tif]

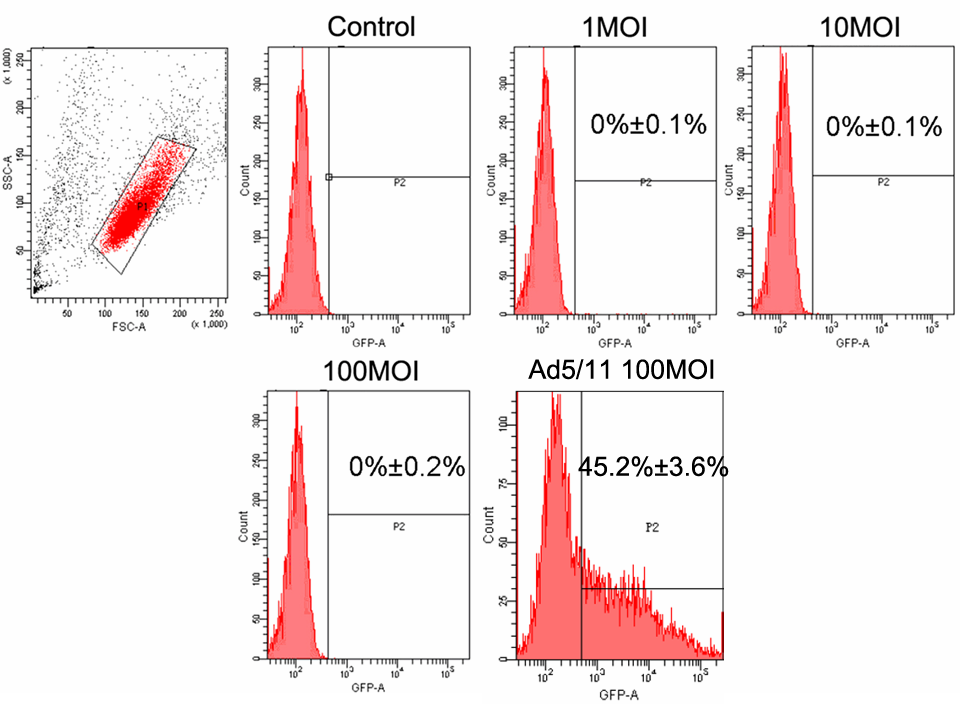

Supplement: Figure S2 — The infection of Ad5-GFP virus in CIK cells. The GFP positive rate was below 0.2% even in 100 MOI. Chimeric adenovirus vector Ad5/11-GFP used as a positive control. The data was presented as mean ± SD of three independent experiments. (TIF) [file pone.0044802.s002.tif]

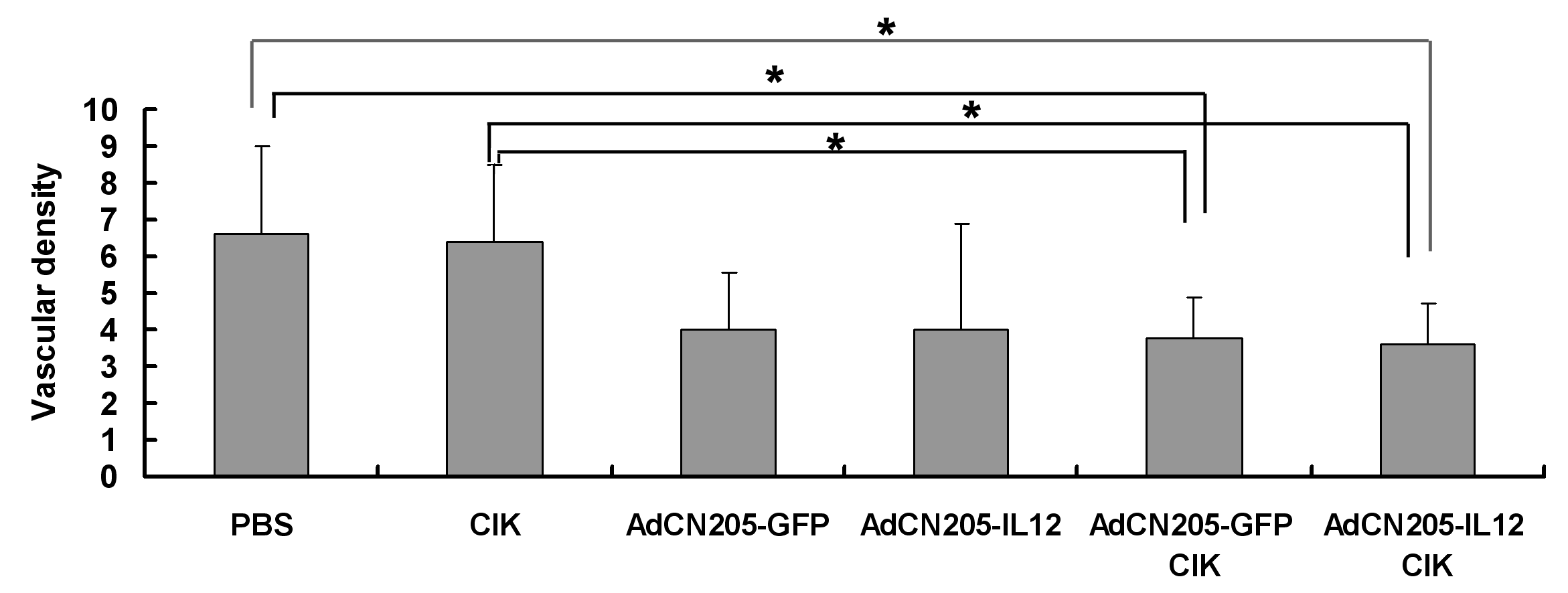

Supplement: Figure S3 — The vascular density in tumor. The vascular density was quantified by counting numbers of endothelial cells in 5 random fields per section at 200×magnification. The vascular density from PBS group has significant difference with that from AdCN205-GFP plus CIK (p = 0.045499) and AdCN205-IL12 plus CIK group (p = 0.035946). The vascular density from CIK group has significant difference with AdCN205-GFP plus CIK (p = 0.038171) and AdCN205-IL12 plus CIK group (p = 0.029449). (TIF) [file pone.0044802.s003.tif]

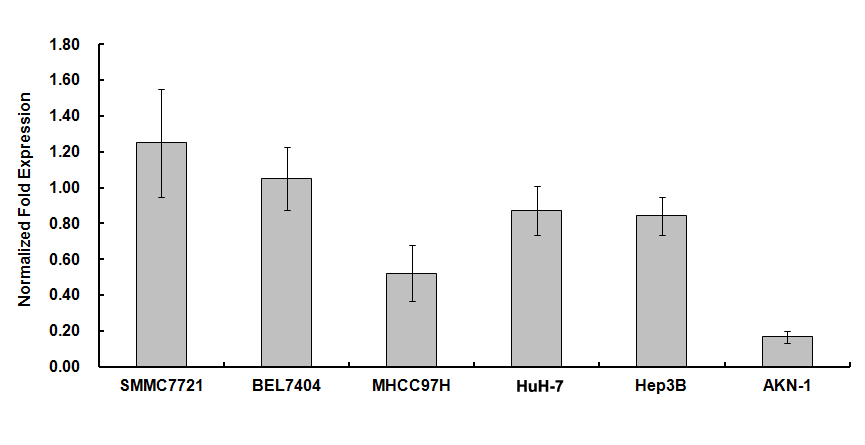

Supplement: Figure S4 — The detection of hTERT expression in liver cancer and normal liver cell lines by real time PCR. The expression of hTERT gene of liver cancer cell lines (SMMC7721, BEL7404, MHCC97H, HuH-7 and Hep3B) and normal liver cell (AKN-1) was detected by real time PCR. (TIF) [file pone.0044802.s004.tif]
